# Does gender equality in sports matter? examining the socio-economic impact on public perceptions

**DOI:** 10.3389/fspor.2025.1537064

**Published:** 2025-02-28

**Authors:** Alessandro Indelicato

**Affiliations:** ^1^ School of Theology, University of Eastern Finland, Joensuu, Finland; ^2^ Departament of Applied Economics, Universidad de Las Palmas de Gran Canaria, Las Palmas de Gran Canaria, Spain

**Keywords:** gender equality attitudes, Fuzzy-Hybrid TOPSIS, LPA, Multinomial Logistic Regression (MLR) models, Eurobarometer

## Abstract

**Introduction:**

Gender equality (GE) is increasingly becoming a key point on modern political agendas. While governments and civil societies strive to achieve this goal, we may be far from “perfect” equality between women and men. Sport is a good example of some of the inequalities that men and women face, such as pay, discrimination, and unequal opportunities.

**Methods:**

The study uses data from the Special Eurobarometer 525 (April–May 2022) to understand attitudes towards GE in sports (ATGEQS). By applying Fuzzy-Hybrid TOPSIS approach, and other methods like Latent Profile Analysis and Multinomial Logistic Regression, I investigate how gender, age, income, education, political beliefs and nationality affect these attitudes.

**Results and Discussion:**

The Nordic countries have the highest ATGEQS, while support for EU GE policies, left-wing views, and life satisfaction is positively related to favourable attitudes. The findings highlight the need for awareness and policies for sports participation to be created, with greater emphasis on disadvantaged groups.

## 1 Introduction

Gender Equality (GE) has thus become central to European political agendas and has inspired several policies: the double gender preference in elections, as explained by Möschel (1); Scandinavian initiatives to integrate mothers into the labour market, by Kjeldstad (2); the recent Spanish app to involve men in sharing housework, according to Ministero de Igualdad (3). GE has been shaped by historical milestones, from the suffragette movement (4) to women's right to vote in the US (5) and the CEDAW convention defining GE and women's rights (6). Despite progress, achieving full GE remains a challenge. While more women hold leadership positions (7, 8), inequalities persist, particularly in wages (9), job opportunities (10), and maternal labour force inclusion (11).

**Figure 1:**
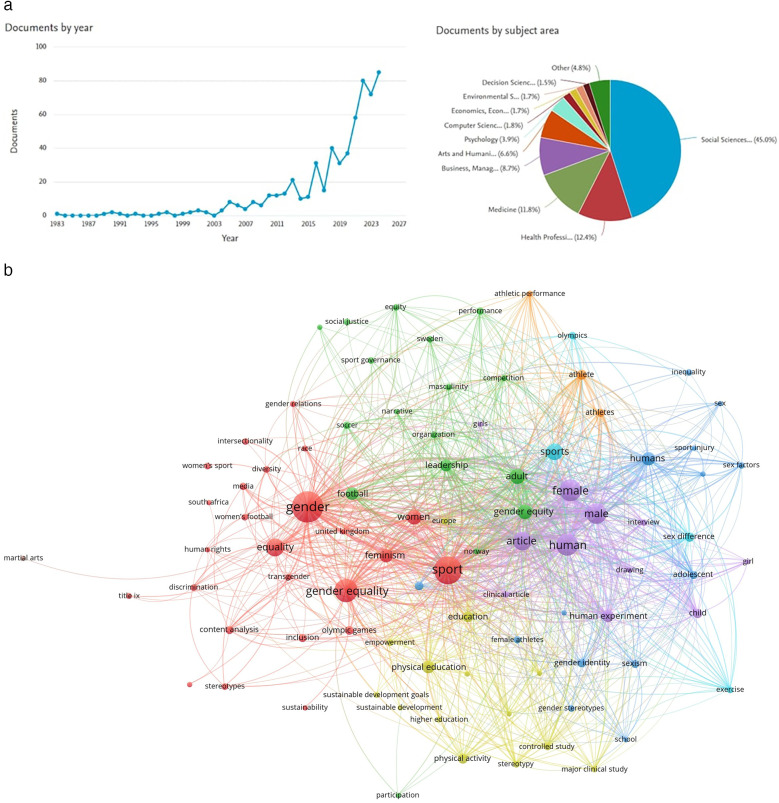
**(a)** Scopus search. **(b)** Topics on Scopus Search.

Beyond economic concerns, gender inequality extends to social roles and sport. Studies show lower parental support for girls' participation in sport (12) and minimal media coverage of women's sport (13). However, successful pop-events such as Spain winning the 2023 Women's World Cup (14) signal progress. Figure 1a shows that there has been a lot more research published in the past decade, particularly from 2015, which suggests that more and more people in academia are interested in GE. Looking at the breakdown by subject area, we can see that there are a lot of studies in the social sciences, health professions and medicine, but not so many in quantitative analysis subjects like decision sciences or economics. This suggests a lack of research methods that focus on statistical analysis and data-driven approaches. Figure 1b shows the most important keywords and topics related to GE in sports. It is dominated by terms such as “gender equality”, “human rights”, and “discrimination”. But there are hardly any keywords referring to quantitative methodology, such as “statistical analysis” or “quantitative research”. This suggests that most studies rely on theoretical ideas rather than real-life, measurable approaches.

The study aims to fill this quantitative gap in the field of GE in sport and provide new insights into the influence of socio-economic factors through data provided by the Special Eurobarometer 525 (April–May 2022). First, Fuzzy-Hybrid TOPSIS is used to provide a Synthetic Indicator of the Europeans' Attitudes Towards Gender Equality in Sport (ATGEQS), then Latent Profile Analysis to cluster respondents based on these attitudes. Finally, Multinomial Logistic Regression is used to analyse the influence of socio-economic factors on the ATGEQS.

The remainder of the paper is structured as follows: Section 2 provides a brief theoretical background on GE in sport, Section 3 describes the data used, while Section 4 examines the methodologies employed. Section 5 analyses the main findings and Section 6 discusses the results. Finally, the paper concludes in Section 7 with final conclusions.

## 2 Gender equality in sports – a brief theoretical overview

In 1994, the Brighton Declaration became a landmark international framework for sport and gender equality, outlining a comprehensive plan that emphasised the full inclusion of women in all aspects of sport and physical activity. The Declaration builds on existing local, national and international regulations, but aims to set a higher standard by promoting global equity in sport. It marks a significant step in challenging gender norms in sport by supporting the representation and active participation of women at all levels (15). Eight years later, the Montreal Toolkit is a practical extension of this vision, providing resources specifically designed to support the role of women in sport through advocacy, leadership and organisational change. The toolkit focuses on cultural and institutional change in the sport sector, emphasising concrete steps to empower women and diversify sport leadership structures (16). Despite these initiatives, studies by Adriaanse and Claringbould (17) and Sheehy and Solvason (18) continue to emphasise the importance of monitoring women's progress in sport to bring about deeper structural change in decision-making processes. This observation is crucial as they highlight the need to include women in leadership as a catalyst for broader change in sport (12, 16–20).

Cultural gender norms are pervasive influences that shape perceptions of which sports are appropriate for women and often limit their participation and acceptance (21). Society tends to categorise sports as either male or female, based on traditional views of gender characteristics. “Masculine” sports, such as football, rugby and boxing, are associated with physical strength, aggression and competitiveness (22–24), while sports considered “feminine”, such as gymnastics and figure skating, are associated with grace, aesthetics and agility (25). These cultural biases influence the acceptance and support of women in different sports, especially those labelled as “masculine” (26). Women involved in football, for example, face social disapproval and prejudice when challenging entrenched gender roles, and therefore the perception that intense contact sports are incompatible with femininity leads to stigmatisation (27). As a result, this social prejudice limits women's participation in certain sporting activities and reduces their recognition as serious athletes in sporting culture (28).

Family and societal expectations also have a significant impact on women's access to sport. Ince-Yenilmez (29) explains that cultural beliefs about gender roles are deeply rooted in families, which often act as gatekeepers to women's participation in sport. Parents may discourage their daughters from participating in sports such as weightlifting because they fear how these activities will affect their femininity and social status (30). Such social norms are particularly influential in more conservative areas, where traditional roles often prioritise domestic “duties” over personal ambitions in competitive sport (31).

Thus, GE in sport faces significant cultural and political challenges that perpetuate inequalities in opportunities, recognition and treatment of male and female athletes (32, 33). Cultural norms and stereotypes strongly influence the participation and perception of women in sport. In many societies, traditional beliefs reinforce the idea that sport is predominantly a male domain. In Ghana, for example, cultural expectations of femininity discourage women from participating in sport because it is seen as a predominantly male activity (34). Similarly, physical education in primary schools often reflects social gender norms, with boys more likely to be encouraged to participate in sports associated with masculinity, such as football, while girls are directed towards less physically demanding activities (35, 103).

Sport funding policies also tend to favour so-called “men's sports”, limiting the resources available to women. According to Druckman and Sharrow (36), the historical underfunding of women's sport, coupled with inadequate political support, exacerbates this inequality. Policy decisions regarding the allocation of resources and support for “women's sport” continue to be heavily influenced by traditional views that favour men's sport on the assumption that it attracts more spectators and revenue. Thus, the intersection of cultural and political barriers is evident in the systemic exclusion of women from leadership roles in sports organisations, perpetuating male-dominated decision-making structures.

Another key aspect of gender inequality in sport is media coverage. Indeed, there is often a tendency to objectify female athletes, emphasising their physical appearance rather than their skills or competitive achievements (37). In this regard, Harmon (38) notes that media coverage of female athletes often emphasises attributes related to beauty and family roles, downplaying their athletic contributions. This objectification reinforces the stereotype that women must conform to traditional standards of femininity, discouraging younger generations from participating in sport and influencing public perceptions of female athletes (39). This bias limits sponsorship opportunities for women, as companies tend to invest in athletes who are publicly recognised for their achievements, a recognition often reserved for men in male-dominated sports. Furthermore, O'neill and Mulready (40) find that women's sports receive significantly less media coverage than men's sports, contributing to the invisibility of female athletes and reducing their potential for sponsorship and support. Where women's sport is included, it tends to focus on traditional narratives that reinforce gender stereotypes, rather than the skills and achievements of female athletes (41). This media exclusion not only affects the visibility of female athletes, but also contributes to a cycle of underrepresentation that affects the development of role models for young girls who aspire to participate in sport.

Although some countries, such as Spain and Canada, have introduced legislative frameworks to promote inclusivity, the effectiveness of these frameworks often depends on their practical implementation and public support. As in Salazar Benítez (42), Spain has a clear legislative basis to support GE in line with international and EU directives, albeit still fragile. However, Pérez-Ugena (43) points out that enforcement in the sports sector is inconsistent, with many sports organisations failing to meet GE standards due to a lack of accountability mechanisms. Activists call for stronger regulatory measures, such as mandatory compliance requirements and penalties for non-compliance, for sports organisations to actively promote GE and address inequalities in areas such as funding and media coverage. On the other hand, other policies on gender inclusion in sport, such as in the case of Canada, reflect avant-garde intentions with commitments such as achieving GE by 2035 (44). However, as Harmon (38) notes, these policies often remain aspirational without being effectively implemented at the local level. Indeed, local sport organisations face challenges such as insufficient funding, limited awareness and cultural resistance that prevent policies from being translated into concrete actions. As a result, while frameworks exist to support women's participation in sport, the actual representation of women in these positions remains low, highlighting the need for more targeted efforts and resources to bridge the gap between policy and practice (45). The first hypothesis is therefore as follows, given that anthropological, cultural and political profiling is crucial to understanding GE in sport:

*H*_1_ = *There are differences in Gender Equality Perception across European Countries*.

While social and cultural structures may influence individual attitudes toward GE in sport, these perceptions may also be significantly shaped by personal socio-economic factors. The interplay between socio-economic factors and GE in sport participation is multifaceted, with different dimensions shaping accessibility, governance, cultural acceptability and professional viability. For example, research has shown that gender quotas in sport governance structures help to increase women's representation in leadership positions, but their effectiveness depends on broader organisational and cultural changes (46). Similarly, socio-economic status (SES) is seen as a key determinant of access to sports facilities. For example, communities with higher SES have better infrastructure and therefore more opportunities for sports participation (47). The economic divide reinforces the impact of gender inequalities in access for sportswomen from low SES backgrounds. At the youth level, children from low-income families are less likely to specialise in sport at an early age, further limiting girls' opportunities to develop a sporting career (48). Thus, the second hypothesis is proposed as follows:

H_2_ = *Socioeconomic factors play a fundamental role in shaping these attitudes*.

Despite the extensive academic focus on gender inequality in sport, this literature review reveals significant methodological gaps. Adriaanse and Schofield (46) identify a key gap in quantitative research on GE. While there are some qualitative findings suggesting that policies may act as a catalyst for change, without quantitative measures of their impact on participation rates and decision-making processes, the true effectiveness of these cannot be determined. Their study calls for more extensive quantitative research to determine how the quota system contributes to measurable variables that may change, such as the number of women in leadership positions and their ability to make and influence organisational policy. Building on this, the third hypothesis suggests that innovative methods, such as Fuzzy-Hybrid TOPSIS and Multinomial Logistic Regression Models (that will be detailed in Section 4), may reveal variations in GE perceptions across different cultural and national contexts. Thus, the second hypothesis is therefore structured as follows:

*H*_2_ = *New methodological approaches in the field can provide consistent insights in GE in Sport*.

## 3 Data

The study analyses data from the Special Eurobarometer 525 “Sport and Physical Activity”, conducted between April and May 2022 and published in September 2022. It is a survey commissioned by the European Commission's Directorate-General for Education, Youth, Sport and Culture (DG EAC) and carried out by the Kantar network through face-to-face and online interviews in the 27 countries of the European Union (EU). A total of 26,580 responses were collected, covering various social and demographic segmentations. As a part of the goals of the European Union Work Plan for Sport, DG EAC, this Eurobarometer tents to “*promoting good governance including the safeguarding of minors, taking account of the specificity of sport, combatting corruption and match fixing, and fighting doping*”, explore “*the economic dimension of sport, in particular innovation in sport, and sport and the digital single market*”, and to promote “*social inclusion, the role of coaches, education in and through sport, sport and health, sport and environment, sport and media and sport diplomacy*” (49, p. 4–5).

The sample is well represented across the 27 EU countries, falling within the 95% confidence interval for representativeness, as well as for other segmentation groups such as age and gender. Almost 60% of respondents are fairly satisfied with their lives, more than 50% are married and more than 42% of the sample never exercise. Most respondents have been studying for more than 16 years and only 7.48% are still studying, while almost 30% are already retired. In addition, almost 70% live in small towns or villages and 25.25% have some difficulty paying their bills. 21% of the sample are manual workers, while more than 25% are managers or other white-collar workers. The majority of respondents do not believe that GE is the European Parliament's (EP's) top priority in terms of values and policies, fighting discrimination and promoting diversity in society. See Table A1 for more details.

**Figure 2:**
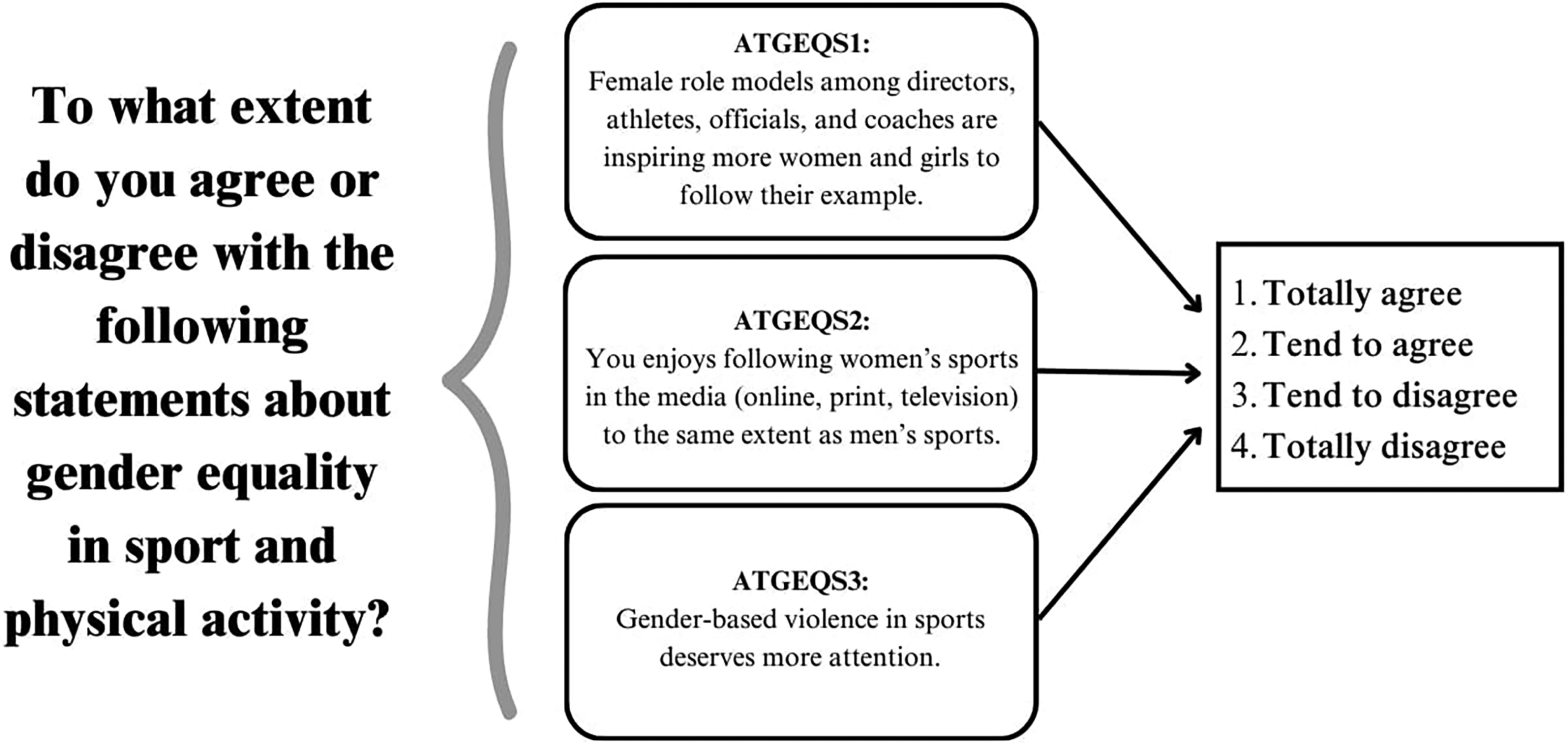
Items.

This special Eurobarometer fits the goal of the paper, as explore the role of Gender Equality in sport and physical activity by measuring Europeans' knowledge and attitudes towards Gender Equality in sport. Respondents are asked to give their opinion on the role of women as role models in sport, the extent to which women's sport is covered in the media and, finally, their personal perception of gender violence in sport. In order to measure Europeans' attitudes towards gender equality in sport (ATGEQS), the study therefore considers these three different items. The survey uses a Likert scale from 1 (strongly agree) to 4 (strongly disagree) to record the level of agreement with these three statements (see Figure 2). However, to facilitate analysis of the items, the scale is inverted so that higher scores represent more “positive” attitudes.

## 4 Methodology

### 4.1 Fuzzy-Hybrid TOPSIS

Surveys are often a good tool for studying socio-economic phenomena, as they provide information on citizens' opinions on a specific issue (50). In the case of the study, the items selected for the analysis of the ATGEQS come from three different statements on a four-point Likert scale. This method of capturing opinion through scales is widely used by researchers when constructing a latent variable (LV) to analyse a socio-economic phenomenon, where each respondent indicates the “degree of agreement” with each statement (51). Methods for analysing this type of information, such as principal component analysis, factor analysis or structural equation models, are often used (52–54). However, these approaches have also been criticised by researchers who argue that their implementation results in the loss of a great deal of information and does not take into account the subjectivity of the answers given by respondents (55). For this reason, the study proposes an alternative approach based on mathematical deterministic methods, such as fuzzy logic, and multi-criteria decision making (MCDM) tools, such as the Technique for Order of Preference by Similarity to Ideal Solution (TOPSIS), to provide a synthetic indicator (SI) capable of measuring Europeans' ATGEQS.

Zadeh (56) is the pioneer of Fuzzy Logic, as try to overcome the limitation of Boolean logic of true or false, implementing an approach of processing values able to allow an interval of possible truth values that can be processed in the same variable. Thus, this approach aims to solve problems when scientists analyse imprecise spectrum of data, providing tools to obtain a set of accurate conclusions (57). There are several approaches to manage the imprecision of the information provided by subjective interview responses, such as using *fuzzification* of the raw information in Triangular Fuzzy Numbers (TFNs) (58), which consists of converting all inputs (original data) into fuzzy membership functions that follow a 3-tuple of values $\left(a_{1},\;a_{2},\;a_{3}\right)$ of TFN as follows:

(1) $$\mu_{A}(x)=\;\{\begin{array}{c} \frac{x-a_{1}}{a_{2}-a_{1}}\;\;\;\;\;a_{1}\leq x\leq \;a_{2}\\ \frac{x-a_{3}}{a_{3}-a_{2}}\;\;\;a_{2}\leq x\leq \;a_{3}\\ 0\;\;\;\;\;\;\;\;otherwise \end{array}$$

The 3-tuple of values $\left(a_{1},\;a_{2},\;a_{3}\right)$ of the TFN for each point of the inverted Likert scale is chosen according to previous studies (59) in the scientific literature (as detailed in Table 1). Overlapping TFNs are an effective tool for smoothing the jump between different fuzzy sets, allowing for gradual membership rather than hard boundaries (60). This reflects real-world scenarios where individual perceptionof two different scale points, such as “tend to disagree” and “disagree”, are often not the same across different sets of respondents (61).

**Table 1:** Triangular fuzzy numbers.

Liker-scale (inverted)	Triangular fuzzy number
1 (Totally disagree)	(0, 0, 50)
2 (Tend to disagree)	(30, 50, 70)
3 (Tend to agree)	(50, 70, 90)
4 (Totally agree)	(70, 100, 100)

Therefore, to analyse each segment of the population, the TFNs are aggregated by average using Fuzzy Set Logic Algebra. However, even though the data are now able to cope with the vagueness and uncertainty of the raw information, they are still difficult to analyse. Therefore, following Kaufmann and Gupta (62), the aggregated TFNs are *defuzzified* by giving more weight to the central values which, according to fuzzy theory, contain more truth. The defuzzified values are thus obtained as follows:

(2) $$v_{\tilde{A}}=\frac{\left(a_{1}+2a_{2}+a_{3}\right)}{4}$$

Following Kaya and Kahraman (63), once information is converted and defuzzified into crisp values ($v_{\tilde{A}}$), TOPSIS steps can be applied to obtain a synthetic indicator capable of measuring the Europeans’ ATGEQS. First, Positive Ideal Solutions (PIS) and Negative Ideal Solutions (NIS) are calculated as the maximum and minimum values, respectively, across the segmentation group for each analysis item, as follows:

(3a) $$PIS_{j}=\{(maxV_{ij}),\;j=1,\;2,\;\ldots n\},\;i=1,2,\;\ldots ,\;m$$

(3b) $$NIS_{j}=\{(minV_{ij}),\;j=1,\;2,\;\ldots n\},\;i=1,2,\;\ldots ,\;m$$

where *V_ij_* are the crisp values obtained by Equation (2), for each group (i=1,2,…,m), and for each item (j=1,2,…,n) (64).

As in Arman et al. (65), the distances between each crisp values and the two ideal solutions can now be calculated using the Euclidean method as follows:

(4a) $$D_{i}^{+}=\;\sqrt{\overset{J}{\underset{\;j=1}{\sum }}\;{\left(PIS_{i}-V_{ij}\right)}^{2}}$$

(4b) $$D_{i}^{-}=\;\sqrt{\overset{J}{\underset{\;j=1}{\sum }}\;{\left(NIS_{i}-V_{ij}\right)}^{2}}$$

As the TOPSIS approach assumes that the best “solution”, in the case of this study the most positive attitude, must be more similar to the PIS and less similar to the NIS (66). Thus, the synthetic indicator measuring the ATGEQS for each segment group of analysis is given by:

(5) $$ATGEQS_{i}=\frac{D_{i}^{-}}{D_{i}^{+}+D_{i}^{-}}\;\to [0,1]$$

The logic behind this indicator is simple. The closer the ATEGQS values are to 1, the more positive the attitudes of Europeans towards GE in sport.

### 4.2 Latent profile analysis

Latent profile analysis (LPA) is a common tool used by quantitative researchers when they want to group, for example, respondents into different clusters based on similarity on a set of variables. It models categorical latent variables that identify defined subpopulations within a population in a defined set of variables (67). Thus, individuals are categorised according to their likelihood of belonging to one cluster or another, generating different profiles based on different characteristics, such as socio-economic.

Unlike other clustering techniques such as k-means or hierarchical clustering, LPA treats profile membership as an unobserved categorical variable. This variable indicates the probability that an individual belongs to a particular profile (68). LPA includes the classification of individuals into clusters based on estimated membership probabilities, the inclusion of different types of variables, and the use of demographics and covariates to describe profiles (69). Thus, this approach focuses on identifying and comparing patterns of variables, allowing the identification of individuals with similar variable patterns and the comparison of these patterns in relation to predictors and outcomes.

To obtain the optimal number of profiles, the algorithm compares results with different numbers of clusters (e.g., 1 cluster, 2 clusters, 3 clusters, etc.) using model selection criteria that penalise overly complex models (i.e., those with too many classes) to avoid overfitting. Following Spurk et al. (67), the study uses Akaike Information Criterion (AIC), Bayesian Information Criterion (BIC) and Entropy to evaluate the best number of profiles for analysis, as follows:

(6) AIC=−2log(L)+2p

(7) BIC=−2log(L)+2log(N)

(8) $$Entropy=1-\frac{-\sum_{i=1}^{N}\;\sum_{c=1}^{C}\;P(C=c|X_{i})log(P(C=c|X_{i}))}{Nlog(C)}$$

where *N* is the sample-size, *p* is the number of parameters in the model, *L* is the likelihood, *C* is the number of latent profiles, and P(C=c∣Xi) is the posterior probability that individual *i* belongs to profile *c*, given their observed data *Xi.* The optimal number of profiles is defined by lower values of AIC and BIC, while higher values of Entropy.

Once the number of best-fitting profiles has been determined, the probability that each individual *i* belongs to cluster c is calculated using Bayes' theorem as follows:

(9) $$P(C=c|Xi)=\;\frac{\pi_{c}\cdot f(X_{i}|\mu_{c},\sigma_{c})}{\sum_{c=1}^{C}\;\pi_{c}\cdot f(Xi|\mu_{c},\sigma_{c})}$$

where $\pi_{c}$ refers to the proportion of individuals in class *c*, and $f(X_{i}|\mu_{c},\sigma_{c})$ stands for the probability density function for the set of observed data *X_i_* given the parameter in the profile *c*. This creates a new variable (Profile) for further analysis. This variable indicates whether an individual belongs to one profile or another.

### 4.3 Multinomial logistic regression

One of the most commonly used methods in the social sciences to analyse the influence of one variable (or set of variables) on another variable is OLS, or when working with latent variables, it is common to use Structural Equation Models (SEM). However, the first method is not feasible in the study because it involves a categorical dependent variable and OLS assumes that the variable under study must be continuous. Also, according to previous research, the SEMs model is skipped because it falls into the loss of too much information (70).

For this reason, the study applies the Multinomial Logistic Regression (MLR) model to manage the categorical nature of the variable obtained by the LPA (profile). The MLR also has other advantages, such as the wide availability of its implementation in almost all statistical software, efficiency and speed in calculating and obtaining results and, finally, ease of interpretation (71).

Following Bansal et al. (72), let *Y* be the dependent variable, which in the case of the study is the “profile”, with *J* categories (where j=1,2,…,J), the probability that observation *i* belongs to category j is given by:

(10) $$P(Y_{i}=j)=\;\frac{e^{X_{i}\beta_{j}}}{1+\sum_{k=1}^{J-1}\;e^{X_{i}\beta_{k}}}$$

where *X_i_* refers to the vector of independent variables (country, age, life satisfaction, education, gender, support for the EP on GE and left-right political self-positioning, among others) and *β_j_* is the vector containing the coefficients for the j-th category.

Usually, when estimating the model, one category (e.g., *j* = 1) is taken as the reference, and this means that the probability of the reference category is evaluated by the probabilities of the other categories (73). Thus, to obtain the estimated coefficients, the MLR compares the log odds of being in category *j* relative to the reference category as follows:

(11) $$ln\left(\frac{P(Y_{i}=j)}{P(Y_{i}=1)}\right)=X_{i}\beta_{j}$$

Thus, each *β_j_* indicates how the log odds of being in category *j* (relative to the reference category, *j* = 1) change for a one-unit increase in the corresponding predictor variable. If it is positive, it indicates an increased likelihood of being in category *j* relative to the reference, while if it is negative, it indicates a decreased likelihood of being in category *j* relative to the reference.

## 5 Results

This section provides the most highlighting insights, using novel quantitative methods in the field of GE in Sport. Firstly, results of applying the Fuzzy-Hybrid TOPSIS are illustrated to get a cross-national overview of the ATGEQS in the European Union (EU). Then, after having clustered individuals into different “profiles”, Multinomial Logistic Regression (MLR) models are implemented to analyse the socioeconomic influence on ATGES.

### 5.1 Exploring gender equality attitudes in sport indicator

**Figure 3:**
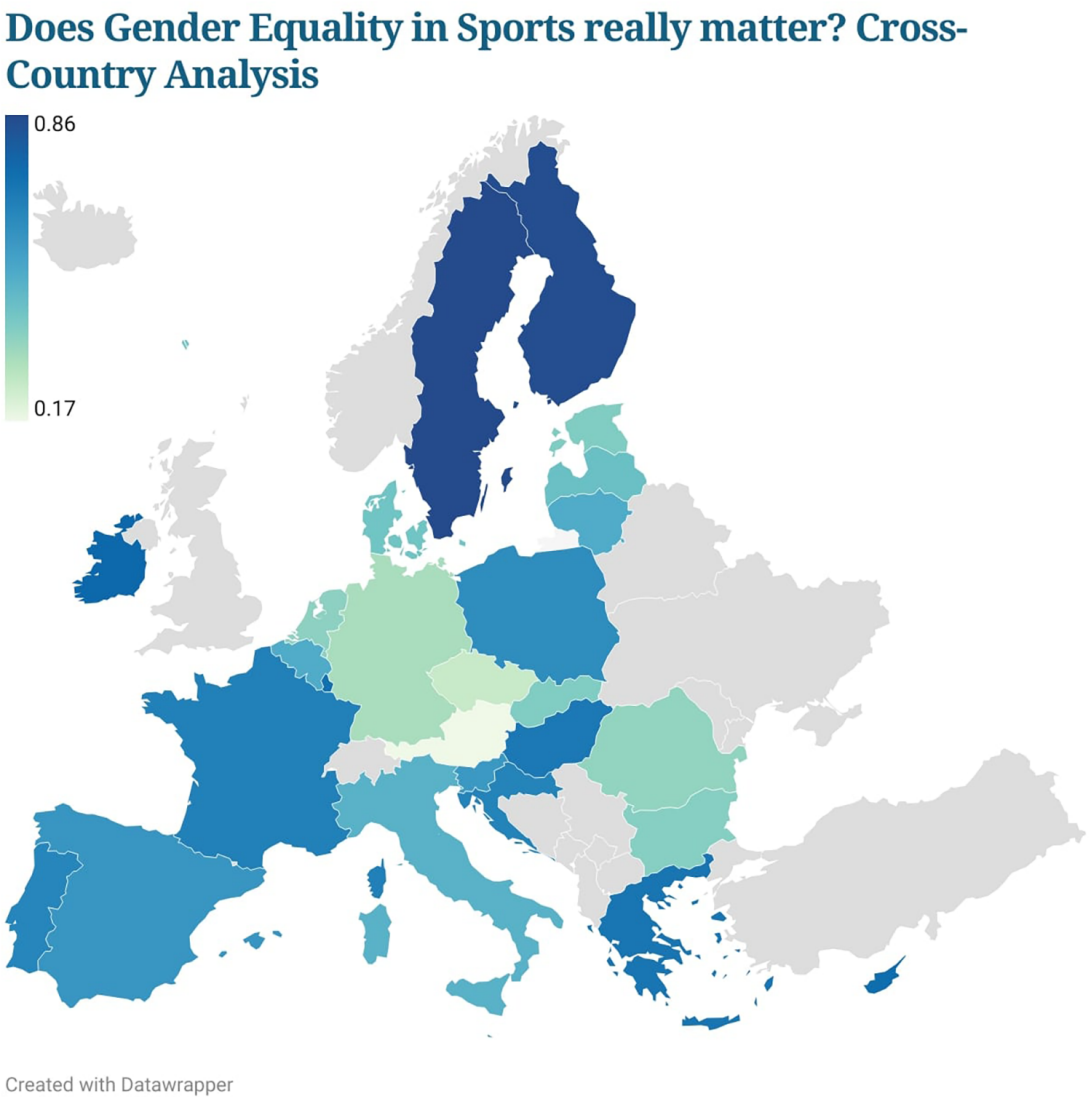
TOPSIS – ATGEQS.

Figure 3 shows the synthetic indicator obtained to measure the ATGEQS (Equations 1–5). The differences between the countries analysed are easily visible, as the map provides an indicative colour legend of the ATGEQS indicator according to a range of values between 0.17, which would indicate the minimum value, and the maximum value (0.86) of the ATGEQS. An analysis of the “different” Europes (74) reveals important differences. The Mediterranean countries present a fairly solid and favourable structure in terms of the perception of GE in sport. Nevertheless, the countries that seem to have higher ATGEQS scores are the northern countries, especially Finland and Sweden.

In contrast, the Central European countries show signs of weakness in recognising the importance of GE in sport. In fact, these countries, led by Austria, have very low ATGEQS scores. This again highlights a regional difference between “different Europeans”. Another significant result concerns the Eastern European countries, led by Poland and Hungary in terms of ATGEQS, while Romania is at the bottom of the ranking with lower scores.

Looking at the details of the differences and analysing all the socio-economic variables, it can be seen that the ideal solutions (PIS and NIS, as in Table 2, Equations 3a,b), formalised by the maximum and minimum values for each item, are occupied only by the country variable. Thanks to this analysis, it is possible to go deeper into the reason for a certain ATGEQS value, as it shows which item is considered more or less important when a nalysing the ATGEQS. Sweden has the highest ATGEQS, and this is mostly because of the role of women in sports management ATGEQS1. This is high-rated because of the country's progressive gender policy and excellent institutional support for equality (75). Finland also has a long history of inclusiveness, anchored in universal suffrage and policies with a gender equity focus (76). On the other hand, Austria, Romania, and the Czech Republic have lower scores because of traditional gender norms (77–79). A lack of interest in women's sports is a big reason why Austria got a low score for ATGEQS2. Romania doesn't put much importance on women in sports management, which is a sign of traditional gender norms. Meanwhile, the Czech Republic doesn't address gender violence in sports, showing a lack of commitment to these issues. Slovenia is focusing on media coverage of women's sports, and Malta is emphasising addressing gender violence.

**Table 2:** PIS and NIS.

Item	Group	PIS^a^	Group	NIS^b^
ATGEQS1	Sweden	84.47	Romania	66.93
ATGEQS2	Slovenia	72.82	Austria	52.70
ATGEQS3	Malta	80.83	Czech Republic	63.41

^a^ Positive ideal solution.

^b^ Negative ideal solution.

### 5.2 Socioeconomic influences on ATGEQS

The use of the LPA technique makes it possible to group Eurobarometer respondents into different “profiles” on the basis of the latent variable and covariates. This method generates a new variable, called “profile”, which indicates which profile an individual is most likely to be associated with. Table 3 gives an overview of the results of the AIC, BIC and Entropy tests (Equations 6–8), which provide important indications of the goodness of the model. According to the literature, lower values of AIC and BIC indicate a better fit of the model, while a higher entropy indicates a clearer classification (67). After careful analysis of different models with different numbers of clusters, the model with the lowest AIC and BIC values, together with a relatively high entropy, is the one that identifies three distinct profiles. Nevertheless, a critical point emerges: the third profile shows a certain weakness, with an entropy of 0.51, indicating less consistency in its definition compared to the other profiles.

**Table 3:** LPA indicators.

Class	AIC	BIC	Entropy
1	−11,333	−11,317	1.00
2	−14,319	−14,286	0.96
3	−14,367	−14,318	0.51

**Figure 4:**
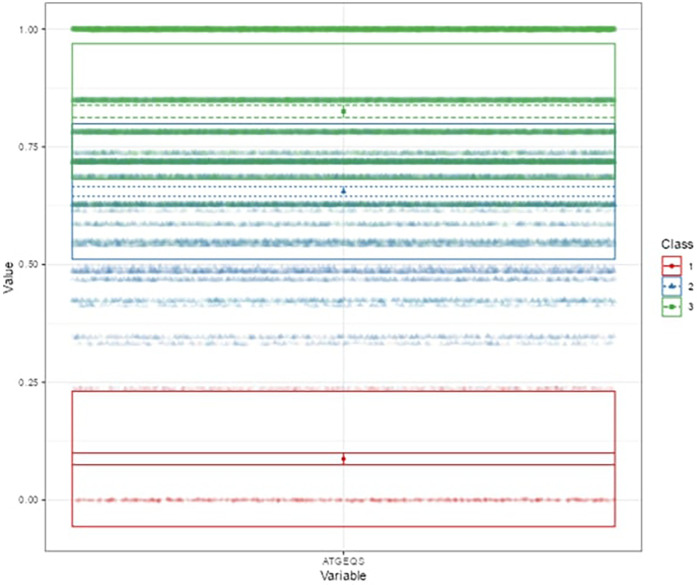
Latent profile analysis.

Figure 4 shows the density distributions and the position of each profile (Equation 9). The results show three distinct profiles: Profile 1, characterised by the lowest values, Profile 2, representing the intermediate values, and Profile 3, associated with the highest values. Furthermore, the highest density is found in the intermediate profile. This result opens up interesting reflections on the dynamics and differences between the groups, suggesting a possible polarisation between those at the extreme ends of the spectrum analysed.

In addition, Tables 4, 5 show the results of the MLR model for Profile 3 (Equations 10, 11), which is characterised by high ATGEQS values, compared to Profile 1, which has lower values. To facilitate the understanding of the results, only estimates with statistical significance (*p*-value less than 0.05) are reported. Among the socio-economic factors, political orientation emerges as a crucial predictor in the modelling of the ATGEQS. Respondents who identify themselves politically on the left are more likely to belong to the third profile, characterised by the highest ATGEQS, than to the first profile, while the opposite is true for centre-right voters. Another relevant predictor is life satisfaction: the higher the level of satisfaction, the higher the probability of belonging to the group with the most positive ATGEQS. Regular sporting activity also has a significant impact on ATGEQS scores, as those who participate in sport tend to have more positive attitudes towards GE in sport.

**Table 4:** Multinomial logistic regression – socioeconomics traits.

Predictor	Estimate	SE	*Z*	*p*
Left	0.39	0.11	3.72	<.001
Centre-right	−0.29	0.15	−1.96	0.05
SL - not very satisfied	−0.55	0.12	−4.62	<.001
SL - fairly satisfied	0.30	0.11	2.81	0.01
SL - very satisfied	0.50	0.12	4.04	<.001
Sport activity - regularly	1.86	0.94	1.99	0.05

SL, satisfaction of life.

**Table 5:** Multinomial logistic regression – gender equality traits.

Predictor	Estimate	SE	*Z*	*p*
Intercept	0.42	0.17	2.51	0.01
EP^a^ gender equality (yes)	0.56	0.11	5.23	<.001
EP^a^ no discrimination (yes)	0.29	0.11	2.69	0.01
EP^a^ diversity (yes)	0.46	0.11	4.18	<.001
EP*a priori*ty GE/Disc./Incl. (yes)	0.52	0.12	4.47	<.001
EP^a^ info GE/Disc./Incl. (no)	0.76	0.13	5.71	<.001
EP^a^ info GE/Disc./Incl. (yes)	1.01	0.20	4.98	<.001
Support GE in sport org. – (yes)	1.23	0.40	3.08	0.00
Contact gender disc. In sport org (yes, management)	1.07	0.49	2.19	0.03

^a^ EP, European parliament.

Finally, the section concludes with an in-depth analysis of the perception of GE and the importance of issues such as non-discrimination, inclusion and the promotion of diversity. The analysis examines whether these issues are perceived as social and political priorities, as well as within the awareness-raising policies promoted by the European Parliament. As might be expected, those who support policies and the dissemination of information on these issues, and who see GE as *a priori*ty for the European Parliament, are more likely to fall into the third profile, reflecting generally more positive attitudes towards GE in sport. Another relevant finding concerns awareness of the existence of information points against gender discrimination in the workplace, as those who are aware of them are significantly more sensitive to the issue of GE in sport. This suggests that access to targeted information can play a key role in promoting positive ATGEQS.

## 6 Discussions

The results of this study indicate large regional and socio-political differences in attitudes towards gender equality in sport, confirming and extending previous research. The high ATGEQS scores in the Nordic countries, particularly Finland and Sweden, are indeed in line with the existing literature, which associates such attitudes with strong welfare policies, progressive gender norms and high levels of female representation in leadership positions (80–84). However, while these findings are consistent with previous research, a closer look reveals that high ATGEQS do not necessarily translate into equal funding in sport, media coverage and pay (104). The above findings also suggest that while women should theoretically be treated equally, women still lag far behind men in coaching and even administrative roles, based on research in countries such as the Nordic states (44).

Conversely, Austria, the Czech Republic and Romania had much lower ATGEQS scores, suggesting that resistance to gender equality in sport is much stronger in these countries. This would be consistent with studies that point to the importance of very strong traditional gender roles, a history of conservative religiosity and older socio-political structures in explaining very low regional support for progressive gender issues (85–87). However, some caution is needed: although religion and conservatism accounted for some of this opposition, the legacy of post-communist economic reform was also crucial. As previous research has shown, the economic upheavals that followed the transition to market economies deprioritised social reforms, including gender equality initiatives (105). This suggests that policy interventions should not only focus on ideological resistance but also address economic constraints that limit institutional support for gender equality in sport.

Religious influences on gender attitudes in sport remain a complex and under-researched area. According to Inglehart (88) and Tröhler (89), historical secularisation in the Nordic countries has favoured greater gender equality in public life, while Catholic and Orthodox traditions in Central and Eastern Europe still uphold patriarchal norms (90). However, recent research suggests that migration and multiculturalism may be reshaping gender dynamics in sport, as has recently been discussed in France (91, 92). Although migration brings new impulses to existing views on gender roles, it can also reignite cultural tensions between traditional values and inclusion policies in sport. The complex interplay of secularism, immigration and gender norms calls for more empirical research in light of ongoing political debates about multiculturalism and integration in European sports institutions.

This study also found a clear ideological divide in the ATGEQS, with left-wing respondents showing greater support for gender equality in sport than their right-wing counterparts. This finding follows wider political trends in which left-wing parties have more frequently aligned themselves with feminist movements, and rapid social change has been framed by conservative parties as an affront to cultural and national identity (93–95). However, such politicisation opens up a critical consideration of the direction in which the future of gender equality in sport may be heading. Although feminist-driven policies have been institutionalised in Western Europe, the rise of nationalism and religious ideologies in some parts of Eastern Europe following the collapse of communism has led to hostility towards many gender equality initiatives (96, 97).

These findings provide a more critical contrast to previous work in which political ideology influences, but is not the sole determinant of, attitudes towards gender equality in sport. Further development is needed on the ways in which economic priorities, media narratives and grassroots activism shape public perceptions. Recent political developments, such as the Spanish debate on the Trans Law, are another area where gender equality policy is increasingly at odds with sport policy itself (95). This divide calls for strategic engagement by political thinkers and sports organisations, so that gender equality in sport is not seen as a partisan issue, but as a core aspect of social justice.

The direct relationship between sports participation and support for GE in sport suggests that the more people are exposed to sporting environments, the more aware they become and the more likely they are to support GE. This finding is consistent with the #MeToo and #SeAcabó movements, which have raised public awareness of gender discrimination in sport (98–100). However, it is important to look critically at the limitations of these movements. While they have been successful in bringing gender inequalities to public attention, institutional responses remain uneven and in some cases performative (101).

Research has shown that awareness campaigns are not enough if they are not accompanied by effective policy enforcement mechanisms (102). For example, the existence of anti-discrimination reporting centres in sports organisations has been reported to be a very effective strategy against harassment and discrimination (101). However, most of them are underfunded and institutionally unbound, and therefore lack significant long-term impact. There is a political imperative to ensure that anti-discrimination and equal pay policies are in place in sport, which can be sustained beyond social movement action and media advocacy.

The findings suggest that both structural and socio-cultural factors are interrelated in achieving gender equality in sport. Increased funding for gender-equitable programmes in sport, with better access for more socio-economic groups, would increase participation. Targeting economic incentives such as scholarships and reduced training fees, may be necessary to address existing inequalities. Greater investment, also, in women's sports infrastructure and media presence could also help to reduce some of the historical inequalities. Conscious public support, facilitated by awareness-raising events that focus on sporting achievement rather than overcoming gender tropes, can also help. Balanced media coverage and improved mechanisms for reporting discrimination and harassment would complement such a policy framework. However, the success of these policies depends on their rigorous enforcement and accountability at all levels of sport.

## 7 Conclusions

The article explores the geographical, social and political differences in Europe on the issue of GE in sport, analysing the countries of the European Union (EU). Using data from the Special Eurobarometer 525 (2022), the analysis adopts the Fuzzy-Hybrid TOPSIS approach to generate a synthetic indicator of attitudes towards gender equality in sport (ATGEQS). The study also identifies the main determinants of such attitudes using latent profile analysis and multinomial logistic regression.

The results underline remarkable geographical differences: the Nordic countries, led by Sweden and Finland, show positive ATGEQS scores, while Austria and Eastern European countries tend to resist progressive values and maintain a more traditional and conservative view of gender roles. Similarly, supporters of policies that promote gender equality within the EU are more likely to belong to the third profile, which is associated with positive attitudes towards gender equality in sport. Awareness of the existence of information points against gender discrimination in the workplace correlates with greater sensitivity to gender equality in sport, underlining the crucial role of targeted information. Politically, left-wing respondents are more likely to belong to the third profile, which has the highest ATGEQS scores, while centre-right respondents are more likely to belong to the first profile. Furthermore, a high level of life satisfaction increases the likelihood of belonging to the group with the highest ATGEQS scores.

Despite the innovative contribution of this study, both from a methodological and thematic point of view, it has some limitations. Firstly, its geographical perspective is limited to the EU, thus excluding neighbouring countries such as Albania, Ukraine, Turkey and others. In this respect, an extension to the continental level could provide a more complete picture of territorial, political and religious differences. Due to limited data availability, the analysis refers to one round in 2022, making the study static rather than dynamic. It would be interesting to include more recent data to assess how these attitudes have changed with the emerging recent geopolitical changes.

## Data Availability

The data used in this study are publicly available here: https://europa.eu/eurobarometer/surveys/detail/2668.

## Appendix

**Table A1:** Descriptive statistics.

Variable	Group	*n*	%^a^	Variable	Group	*n*	%^a^
Country	Austria	1,005	3.78	EP policy info needed: GE	No	23,421	88.15
	Belgium	1,101	4.14		Yes	3,148	11.85
	Bulgaria	1,039	3.91	Sport activity	Regularly	1,876	7.06
	Croatia	1,008	3.79		With some regularity	8,300	31.24
	Cyprus	503	1.89		Seldom	5,205	19.59
	Czech Republic	1,073	4.04		Never	11,161	42.01
	Denmark	1,004	3.78	Support of GE in sport org.	Yes	4,060	15.28
	Estonia	1,030	3.88		No	2,873	10.81
	Finland	1,004	3.78	Know contact to speak to in sport org when Gend. Discr.	Yes, in management	3,449	12.98
	France	1,012	3.81		Yes, a single contact point	1,078	4.06
	Germany	1,511	5.70		No	2,818	10.61
	Greece	1,014	3.82	Left-right placement	(1-2) Left	2,151	8.10
	Hungary	1,025	3.86		(3-4)	4,879	18.36
	Ireland	1,011	3.81		(5-6) Centre	9,874	37.16
	Italy	1020	3.84		(7-8)	4,780	17.99
	Latvia	1,013	3.81		(9-10) Right	1,764	6.64
	Lithuania	1,002	3.77	Marital status	Married	13,773	51.84
	Luxembourg	502	1.89		Single living with a partner	2,690	10.12
	Malta	504	1.90		Single	5,306	19.97
	Poland	1,013	3.81		Divorced or separated	2,126	8.00
	Portugal	1005	3.78		Widow	2,537	9.55
	Romania	1,057	3.98		Other	1,10	0.41
	Slovakia	1,010	3.80	Gender	Man	12,331	46.41
	Slovenia	1,022	3.85		Woman	14,220	53.52
	Spain	1,006	3.79		Other	18	0.07
	Sweden	1,043	3.93	Occupation	Self-employed	1,925	7.25
	The Netherlands	1,032	3.88		Managers	3,144	11.83
Age	15-24	2,381	8.96		Other white collars	3,759	14.15
	25-34	3,332	12.54		Manual workers	5,590	21.04
	35-44	4,127	15.53		House persons	1,175	4.42
	45-54	4,481	16.87		Unemployed	1,093	4.11
	55-64	4,828	18.17		Retired	7,895	29.72
	65+	7,408	27.88		Students	1,988	7.48
Life Satisfaction	Very satisfied	6,363	23.95	Type of community	Rural area or village	8,823	33.21
	Fairly satisfied	15,789	59.43		Small/middle town	9,643	36.29
	Not very satisfied	3,650	13.74		Large town	8,100	30.49
	Not at all satisfied	735	2.77	Difficulties paying bills	Most of the time	1,994	7.50
EP values priority: GE	No	2,1545	81.09		From time to time	6,798	25.59
	Yes	5,024	18.91		Almost never/never	17,580	66.17
EP values priority: Fight Discrimination	No	23,333	87.82	Age education	15-	2,938	11.06
	Yes	3,236	12.18		16-19	11,085	41.72
EP values priority: Diversity in society	No	22,576	84.97		20+	9,723	36.6
	Yes	3,993	15.03		Still Studying	1,988	7.48
EP policy priorities: GE	No	23,139	87.09		No full-time education	181	0.68
	Yes	3,430	12.91				

^a^ Some segment do not reach 100% because of missing values.

## References

[1] Möschel M. The Italian Government Enforces Gender Parity in Regional Elections. Berlin: VerfBlog (2020). Available online at: verfassungsblog.de/the-italian-government-enforces-gender-parity-in-regional-elections

[2] Kjeldstad R. Gender policies and gender equality. In: Kautto M, Fritzell J, Hvinden B, Kvist J, Uusitalo H, editors. Nordic Welfare States in the European Context. London: Routledge (2001). p. 66–8.

[3] Ministerio de Igualdad. Igualdad presenta “MeToca”, la aplicación para repartir de forma corresponsable las tareas domésticas y de cuidados. Ministerio de Igualdad (2023). Available online at: https://www.igualdad.gob.es/comunicacion/notasprensa/igualdad-presenta-metoca-la-aplicacion-para-repartir-de-forma-corresponsable-las-tareas-domesticas-y-de-cuidados/ (Accessed November 10, 2024).

[4] McCammon H Campbell KE. Winning the vote in the west. The political successes of the women’s suffrage movements, 1866–1919. Gend Soc. (2001) 15(1):55–82. 10.1177/089124301015001004

[5] Johnson JM. Not as a favor, not as a privilege, but as a right": woman suffragists, race, rights, and the nineteenth amendment. West N Engl Law Rev. (2020) 42(3):385–97. Available online at: https://digitalcommons.law.wne.edu/lawreview/vol42/iss3/4

[6] Orta LCG. The convention on the elimination of all forms of discrimination against women (CEDAW): from its radical preamble to its contemporary intersectional approach. Womens Hist Rev. (2023) 34(1):79–92. 10.1080/09612025.2023.2277490

[7] Saha S Weeks AC. Ambitious women: gender and voter perceptions of candidate ambition. Polit Behav. (2022) 44(2):779–805. 10.1007/s11109-020-09636-z

[8] Biswas PK Roberts H Stainback K. Does women’s board representation affect non-managerial gender inequality? Hum Resour Manage. (2021) 60(4):659–80. 10.1002/hrm.22066

[9] Petrongolo B Ronchi M. Gender gaps and the structure of local labor markets. Labour Econ. (2020) 64:101819. 10.1016/j.labeco.2020.101819

[10] Le Barbanchon T Rathelot R Roulet A. Gender differences in job search: trading off commute against wage. Q J Econ. (2021) 136(1):381–426. 10.1093/qje/qjaa033

[11] Cortés P Pan J. Children and the remaining gender gaps in the labor market†. J Econ Lit. (2023) 61(4):1359–409. 10.1257/jel.20221549

[12] Women in Sport. Sport, Stereotypes and Stolen Dreams why Girls Still Feel They Don’t Belong in Sport. London: Women in Sport (2023).

[13] UNESCO. Lighting the Torch for Gender Equality in Sports. Paris: United Nations Educational, Scientific and Cultural Organization (2024). Available online at: https://www.unesco.org/en/articles/lighting-torch-gender-equality-sports

[14] Blanco-Gómez ML Gómez-Ortiz MJ. An analysis of linguistic resources for sports tourism Media discourse: a case study of the women’s world cup final in Sydney. Rev Alicant Estud Ingl. (2024) 41:239–60. 10.14198/raei.2024.41.11

[15] Bringhton Declaration. Brighton Declaration on Women in Sport. Brighton, UK: International Working Group on Women and Sport (1994).

[16] Adriaanse J. Gender diversity in the governance of sport associations: the Sydney scoreboard global Index of participation. J Bus Ethics. (2016) 137(1):149–60. 10.1007/s10551-015-2550-3

[17] Adriaanse JA Claringbould I. Gender equality in sport leadership: from the Brighton declaration to the Sydney scoreboard. Int Rev Sociol Sport. (2016) 51(5):547–66. 10.1177/1012690214548493

[18] Sheehy A Solvason C. Teaching lads’ lads and girly-girls: why recognising and tackling gender stereotypes still matters in education. Education. (2023) 51(7):3–13. 10.1080/03004279.2023.2224842

[19] Vicente-Pedraz M Paz Brozas-Polo M. Exo y género en la contienda identitaria del deporte: propuesta de un debate sobre la competición deportiva multigénero [sex and gender in the contest of identity in sport: a proposal for a debate on multi-gender sports competition]. Cult Cien Deporte. (2016) 13(35):101–10. 10.12800/ccd.v12i35.881

[20] Wai LC. Sports, gender and Media: why are there fewer female coaches on the court in Hong Kong? Pak J Life Soc Sci. (2024) 22(2):4152–67. 10.57239/PJLSS-2024-22.2.00307

[21] Jeanes R Spaaij R Farquharson K McGrath G Magee J Lusher D Gender relations, gender equity, and community sports spaces. J Sport Soc Issues. (2021) 45(6):545–67. 10.1177/0193723520962955

[22] Tomlinson A. FIFA. The Men, the Myths and the Money. London, UK: Routledge (2014).

[23] Follmer B Varga AA Zehr EP. Understanding concussion knowledge and behavior among mixed martial arts, boxing, kickboxing, and muay thai athletes and coaches. Phys Sportsmed. (2020) 48(4):417–23. 10.1080/00913847.2020.1729668 32067547

[24] Solomons J Bekker S Groom R Kraak W. Insights into coaching effectiveness: perspectives from coaches and players in South African women’s rugby. Int J Sports Sci Coach. (2025) 20(1):8–21. 10.1177/17479541241283625

[25] Adams ML. The manly history of a “girls” sport’: gender, class and the development of nineteenth-century figure skating’. Int J Hist Sport. (2007) 24(7):872–93. 10.1080/09523360701311752

[26] Bartoluci S Baršić M. “Beautiful, and you play football?”: gender (in)equality and football/futsal. Stud Ethnol Croat. (2020) 32(1):097–126. 10.17234/SEC.32.8

[27] Klenk J. Sport as a strategy to address gender-based violence. [master’s thesis]. Sweden: Jönköping University, Jönköping (2024). Available online at: https://www.diva-portal.org/smash/get/diva2:1876218/FULLTEXT01.pdf

[28] Evans AB Pfister GU. Women in sports leadership: a systematic narrative review. Int Rev Sociol Sport. (2021) 56(3):317–42. 10.1177/1012690220911842

[29] Ince-Yenilmez M. The role of socioeconomic factors on women’s risk of being exposed to intimate partner violence. J Interpers Violence. (2022) 37(9–10):NP6084–111. 10.1177/0886260520966668 33047645

[30] Scraton S. “Boys muscle in where angels fear to tread'–girls” sub-cultures and physical activities. In: Rojek C, Shaw SM, Veal AJ, editors. Sport, Leisure and Social Relations (RLE Sports Studies). London, UK: Routledge (2014). p. 160–86.

[31] Hylton K Totten M. Developing ’Sport for all': addressing inequality in sport. In: Sport Development. London: Routledge (2013). p. 37–79.

[32] Burnett C. Politics of gender (in) equality relating to sport and development within a sub-saharan context of poverty. Front Sociol. (2018) 3:27. 10.3389/fsoc.2018.00027

[33] Adom-Aboagye NAA. Gender Equity in Sport-related policies: A Case Study for Understanding Empowerment and Inclusion in South Africa. South Africa: University of Johannesburg (2020).

[34] Charway D Strandbu Å. Participation of girls and women in community sport in Ghana: cultural and structural barriers. Int Rev Sociol Sport. (2024) 59(4):559–78. 10.1177/10126902231214955

[35] Cárcamo C Moreno A Del Barrio C. Girls do not sweat: the development of gender stereotypes in physical education in primary school. Hum Arenas. (2021) 4(2):196–217. 10.1007/s42087-020-00118-6

[36] Druckman JN Sharrow EA. Equality Unfulfilled: How Title IX’s Policy Design Undermines Change to College Sports. Cambridge: Cambridge University Press (2023).

[37] Kaskan ER Ho IK. Microaggressions and female athletes. Sex Roles. (2016) 74:275–87. 10.1007/s11199-014-0425-1

[38] Harmon SH. Gender inclusivity in sport? From value, to values, to actions, to equality for Canadian athletes. Int J Sport Policy Polit. (2020) 12(2):255–68. 10.1080/19406940.2019.1680415

[39] Darvin L Sagas M. Objectification in sport media: influences on a future women’s sporting event. Int J Sport Commun. (2017) 10(2):178–95. 10.1123/IJSC.2017-0022

[40] O'neill D Mulready M. The invisible woman? A comparative study of women’s sports coverage in the UK national press before and after the 2012 Olympic games. Journal Pract. (2015) 9(5):651–68. 10.1080/17512786.2014.965925

[41] Duncan MC. Gender warriors in sport: women and the media. In: Raney AA Bryant J, editors. Handbook of Sports and Media. New York, NY: Routledge (2009). p. 247–69.

[42] Salazar Benítez O. The fragility of gender equality policies in Spain. Soc Sci. (2016) 5(2):17. 10.3390/socsci5020017

[43] Pérez-Ugena M. Regulatory aspects of the gender issue in sports. Estud Deusto. (2020) 68(2):205–30. 10.18543/ed-68(2)-2020pp205-230

[44] Norman M Donnelly P Kidd B. Gender inequality in Canadian interuniversity sport: participation opportunities and leadership positions from 2010 to 11 to 2016 to 17. Int J Sport Policy Polit. (2021) 13(2):207–23. 10.1080/19406940.2020.1834433

[45] Sotiriadou P De Haan D. Women and leadership: advancing gender equity policies in sport leadership through sport governance. Int J Sport Policy Polit. (2019) 11(3):365–83. 10.1080/19406940.2019.1577902

[46] Adriaanse J Schofield T. The impact of gender quotas on gender equality in sport governance. J Sport Manag. (2014) 28(5):485–97. 10.1123/jsm.2013-0108

[47] Eime R Harvey J Charity M Casey M Westerbeek H Payne W. The relationship of sport participation to provision of sports facilities and socioeconomic status: a geographical analysis. Aust N Z J Public Health. (2017) 41(3):248–55. 10.1111/1753-6405.12647 28110514

[48] Jayanthi N Holt D LaBella C Dugas L. Socioeconomic factors for sports specialization and injury in youth athletes. Sports Health. (2018) 10:303–10. 10.1177/1941738118778510 29851549 PMC6044126

[49] European Commission. Sport and physical activity project title special eurobarometer 525-sport and physical activity. Brussels: European Union (2022). 10.2766/356346

[50] Voas D. Surveys of behaviour, beliefs and affiliation: micro-quantitative. In: The SAGE Handbook of the Sociology of Religion. London: SAGE (2007). p. 144–66. 10.4135/9781848607965.n8

[51] Ben-Akiva M Walker J Bernardino AT Gopinath DA Morikawa T Polydoropoulou A. Integration of choice and latent variable models. In: Perpetual Motion: Travel Behaviour Research Opportunities and Application Challenges. Amsterdam: Elsevier (2002). p. 431–70.

[52] Beattie JR Esmonde-White FW. Exploration of principal component analysis: deriving principal component analysis visually using spectra. Appl Spectrosc. (2021) 75(4):361–75. 10.1177/0003702820987847 33393349

[53] Levy R. Conceptual grounding for Bayesian inference for latent variables in factor analysis. Meas Interdiscip Res Perspect. (2022) 20(4):195–214. 10.1080/15366367.2021.1996819

[54] Feng Y Hancock GR. A structural equation modeling approach for modeling variability as a latent variable. Psychol Methods. (2024) 29(2):262. 10.1037/met0000477 35404625

[55] Martín JC Indelicato A. Comparing a fuzzy hybrid approach with invariant MGCFA to study national identity. Appl Sci. (2023) 13(3):1657. 10.3390/app13031657

[56] Zadeh LA. Fuzzy sets. Inf Control 1965) 8(3):338–53. 10.1016/S0019-9958(65)90241-X

[57] Zimmermann HJ. Fuzzy logic on the frontiers of decision analysis and expert systems. In: Proceedings of North American Fuzzy Information Processing. Piscataway, NJ: IEEE (1996). p. 65–9.

[58] Ahmad Basri NAZ. Construction of fuzzy control charts by using triangular and Gaussian fuzzy numbers for solder paste thickness. [doctoral dissertation, Universiti Tun Hussein Onn Malaysia]. (2018).

[59] Martín JC Indelicato A. A fuzzy-hybrid analysis of citizens’ perception toward immigrants in Europe. Qual Quant. (2023) 57(2):1101–24. 10.1007/s11135-022-01401-0

[60] Kóczy L Zorat A. Fuzzy systems and approximation. Fuzzy Sets Syst. (1997) 85(2):203–22. 10.1016/0165-0114(95)00348-7

[61] Höhne JK Krebs D. Scale direction effects in agree/disagree and item-specific questions: a comparison of question formats. Int J Soc Res Methodol. (2018) 21(1):91–103. 10.1080/13645579.2017.1325566

[62] Kaufmann A Gupta MM. Fuzzy Mathematical Models in Engineering and Management Science. New York, NY: Elsevier Science Inc. (1988).

[63] Kaya T Kahraman C. Multicriteria decision making in energy planning using a modified fuzzy TOPSIS methodology. Expert Syst Appl. (2011) 38(6):6577–85. 10.1016/j.eswa.2010.11.081

[64] Uzun B Taiwo M Syidanova A Uzun Ozsahin D. The technique for order of preference by similarity to ideal solution (TOPSIS). In: Application of Multi-Criteria Decision Analysis in Environmental and Civil Engineering. Cham: Springer (2021). p. 25–30. 10.1007/978-3-030-64765-0_4

[65] Arman H Hadi-Vencheh A Kiani Mavi R Khodadadipour M Jamshidi A. Revisiting the interval and fuzzy TOPSIS methods: is Euclidean distance a suitable tool to measure the differences between fuzzy numbers? Complexity. (2022) 2022(1):7032662. 10.1155/2022/7032662

[66] Wang TC Thu Nguyen TT Phan BN. Analyzing higher education performance by entropy-TOPSIS method: a case study in Vietnam private universities. Meas Control. (2022) 55(5-6):385–410. 10.1177/00202940221089504

[67] Spurk D Hirschi A Wang M Valero D Kauffeld S. Latent profile analysis: a review and “how to” guide of its application within vocational behavior research. J Vocat Behav. (2020) 120:103445. 10.1016/j.jvb.2020.103445

[68] Magidson J Vermunt JK. A nontechnical introduction to latent class models. In: Statistical Innovations White Paper, 1. Belmont, MA: Statistical Innovations Inc. (2002). p. 15.

[69] Collins LM Lanza ST. Latent Class and Latent Transition Analysis: With Applications in the Social, Behavioral, and Health Sciences (Vol. 718). Hoboken, NJ: John Wiley & Sons (2009).

[70] Indelicato A Martín JC. The effects of three facets of national identity and other socioeconomic traits on attitudes towards immigrants. J Int Migr Integr. (2024) 25(2):645–72. 10.1007/s12134-023-01100-1

[71] Kwak C Clayton-Matthews A. Multinomial logistic regression. Nurs Res. (2002) 51(6):404–10. 10.1097/00006199-200211000-00009 12464761

[72] Bansal P Krueger R Bierlaire M Daziano RA Rashidi TH. Bayesian Estimation of mixed multinomial logit models: advances and simulation-based evaluations. Trans Res B Methodol. (2020) 131:124–42. 10.1016/j.trb.2019.12.001

[73] Fox J Andersen R. Effect displays for multinomial and proportional-odds logit models. Sociol Methodol. (2006) 36(1):225–55. 10.1111/j.1467-9531.2006.00180.x

[74] Boatcă M. Multiple Europes and the politics of difference within. In: The Study of Europe. Baden-Baden: Nomos Verlagsgesellschaft mbH & Co. KG (2010). p. 51–66.

[75] Pfister G. Gender equality and (elite) sport. In: Enlarged Partial Agreement on Sport. Strasbourg: Council of Europe (2011). p. 51–66.

[76] Erikson J Björk A Paavola JM. Still signs of masculine norms in the parliamentary workplace? Political gender equality in Finland and Sweden after a century of universal suffrage. In: Suffrage and Its Legacy in the Nordics and Beyond: Gender, Institutional Constraints and Feminist Strategies. Cham: Springer Nature Switzerland (2024). p. 199–220.

[77] Gresch N Sauer B. Debates on Women’s Representation in Austria. Or: The Development of the Pitfalls of a Conservative Gender Regime. Vienna: CADMUS (2015).

[78] Nešporová O. (2019). Non-normative parents in the gender-traditional Czech republic. In: New Parents in Europe. Cheltenham: Edward Elgar Publishing. p. 207–24. 10.4337/9781788972970

[79] Bucur M. Gender analysis and gender ideology: gender studies in Romania. Stud Polit. (2021) 21(2):385–407.

[80] Bergqvist C. Equal Democracies?: Gender and Politics in the Nordic Countries. Copenhagen: Nordic Council of Ministers (1999).

[81] Esping-Andersen G. Incomplete Revolution: Adapting Welfare States to Women’s new Roles. Cambridge, UK: Polity (2009).

[82] Larsen E. The gender-progressive Nordics: a matter of history. In: Gender Equality and Nation Branding in the Nordic Region. New York, NY: Routledge (2021). p. 13–38.

[83] Vehviläinen H Itkonen H Szerovay M Nevala A. Equality and gender. In: Wagner U Jørgensen KFA Persson R, editors. Football in the Nordic Countries. London, UK: Routledge (2023). p. 133–75.

[84] Virkki T. At the interface of national and transnational: the development of Finnish policies against domestic violence in terms of gender equality. Soc Sci. (2017) 6(1):31. 10.3390/socsci6010031

[85] Cummings KS. New Women of the Old Faith: Gender and American Catholicism in the Progressive Era. Chapel Hill, NC: Univ. of North Carolina Press (2009).

[86] Fábián K Johnson JE Lazda M. The Routledge Handbook of Gender in Central-Eastern Europe and Eurasia. New York, NY: Routledge (2021).

[87] Sen G Mukherjee A. No empowerment without rights, no rights without politics: gender-equality, MDGs and the post-2015 development agenda. In: The MDGs, Capabilities and Human Rights. New York, NY: Routledge (2017). p. 92–106.

[88] Inglehart R. Rising Tide: Gender Equality and Cultural Change Around the World. Cambridge, UK: Cambridge University Press (2003).

[89] Tröhler D. The lasting legacy of the European reformation of the 16th century: protestant foundations of modern educational reasoning. J Beliefs Values. (2021) 42(2):258–76. 10.1080/13617672.2020.1818934

[90] Starkey C Tomalin E. The Routledge Handbook of Religion, Gender and Society. London: Routledge (2022).

[91] Glas S. Exclusionary contexts frustrate cultural integration: migrant acculturation into support for gender equality in the labor market in Western Europe. Int Migr Rev. (2022) 56(3):941–75. 10.1177/01979183211059171

[92] Mancini S Cohen EL. Gender justice and religious freedom in the post-secular age. In: Constitutions and Religion. Cheltenham, UK: Edward Elgar Publishing (2020). p. 347–69.

[93] Bernárdez-Rodal A Rey PR Franco YG. Radical right parties and anti-feminist speech on Instagram: vox and the 2019 Spanish general election. Party Polit. (2022) 28(2):272–83. 10.1177/1354068820968839

[94] Hunter ND. In search of equality for women: from suffrage to civil rights. Duq L Rev. (2021) 59:125.

[95] Lavizzari A Pirro AL. The gender politics of populist parties in Southern Europe. West Eur Polit. (2024) 47(7):1473–502. 10.1080/01402382.2023.2246110

[96] Haynes J. Right-wing populism and religion in Europe and the USA. Religions (Basel). (2020) 11(10):490. 10.3390/rel11100490

[97] Liston K Hellstrand L O’Leary C. Public service broadcasting and gender equal coverage: reflections on research and practice in Ireland and Sweden. Sport Soc. (2024) 27(6):860–76. 10.1080/17430437.2024.2334593

[98] Fenton A Ahmed W Hardey M Boardman R Kavanagh E. Women’s football subculture of misogyny: the escalation to online gender-based violence. European Sport Management Quarterly. (2023):1–23. 10.1080/16184742.2023.2270566

[99] Fowler C Moro S. Introduction:#(No) SeAcabó/it is (not) over: the rubiales/hermoso non-consensual kiss and the growth of a culture of concern. Eur J Cult Stud. (2024):13675494241270387. 10.1177/13675494241270387

[100] Horeck T. Sexual violence and social justice: the celebrity# MeToo documentary in the US. In: The Routledge Companion to Gender, Media and Violence. New York, NY: Routledge (2023). p. 232–41.

[101] Hartmann-Tews I. Gender-based violence and organizational silence in voluntary sports organizations. In: Starystach S, Höly K, editors. Silence of Organizations: How Organizations Cover up Wrongdoings. Heidelberg: heiBOOKS (2021). p. 169–92. 10.11588/heibooks.592.c11623

[102] Craig K Liao M. Tackling Violence Against Women and Girls in Sport: A Handbook for Policy Makers and Sports Practitioners. Paris: UNESCO Publishing (2023).

[103] Roberts J Gray S Miñano MJ. Exploring the PE contexts and experiences of girls who challenge gender norms in a progressive secondary school. Curr Stud Health Phys Educ. (2020) 11(1):3–17. 10.1080/25742981.2019.1696688

[104] Pfister G. Sportswomen in the German popular press: a study carried out in the context of the 2011 Women’s Football World Cup. Soccer Soc. (2014) 16(5–6):639–56. 10.1080/14660970.2014.963314

[105] Fodor E. More babies for the state: the “carefare” regime of anti-liberal hungary. New Labor Forum. (2022) 31(1):34–41. 10.1177/10957960211062460

